# Comparing neuronal oscillations during visual spatial attention orienting between normobaric and hypobaric hypoxia

**DOI:** 10.1038/s41598-023-45308-8

**Published:** 2023-10-21

**Authors:** Evan A. Hutcheon, Vasily A. Vakorin, Adonay S. Nunes, Urs Ribary, Sherri Ferguson, Victoria E. Claydon, Sam M. Doesburg

**Affiliations:** 1 Department of Biomedical Physiology and Kinesiology, Simon Fraser University, Burnaby, BC Canada; 2 Department of Neurology, Massachusetts General Hospital, Harvard Medical School, Harvard University, Boston, MA USA; 3 Department of Psychology, Simon Fraser University, Burnaby, BC Canada; 4 Environmental Physiology and Medicine Unit, Faculty of Science, Simon Fraser University, Burnaby, BC Canada; 5 Institute for Neuroscience and Neurotechnology, Simon Fraser University, Burnaby, Canada

**Keywords:** Neuroscience, Physiology

## Abstract

Normobaric hypoxia (NH) and hypobaric hypoxia (HH) are both used to train aircraft pilots to recognize symptoms of hypoxia. NH (low oxygen concentration) training is often preferred because it is more cost effective, simpler, and safer than HH. It is unclear, however, whether NH is neurophysiologically equivalent to HH (high altitude). Previous studies have shown that neural oscillations, particularly those in the alpha band (8–12 Hz), are impacted by hypoxia. Attention tasks have been shown to reliably modulate alpha oscillations, although the neurophysiological impacts of hypoxia during cognitive processing remains poorly understood. To address this we investigated induced and evoked power alongside physiological data while participants performed an attention task during control (normobaric normoxia or NN), NH (fraction of inspired oxygen = 12.8%, partial pressure of inspired oxygen = 87.2 mmHg), and HH (3962 m, partial pressure of inspired oxygen = 87.2 mmHg) conditions inside a hypobaric chamber. No significant differences between NH and HH were found in oxygen saturation, end tidal gases, breathing rate, middle cerebral artery velocity and blood pressure. Induced alpha power was significantly decreased in NH and HH when compared to NN. Participants in the HH condition showed significantly increased induced lower-beta power and evoked higher-beta power, compared with the NH and NN conditions, indicating that NH and HH differ in their impact on neurophysiological activity supporting cognition. NH and HH were found not to be neurophysiologically equivalent as electroencephalography was able to differentiate NH from HH.

## Introduction

Between 1981 and 2003 the United States Air Force has reported 221 instances of hypoxia with three deaths during aircraft depressurisation incidents^1^. High Altitude Indoctrination (HAI) programs are performed so that pilots can become acquainted with their own hypoxic symptoms. This enables them to recognize hypoxia early in cases of sudden aircraft depressurization, facilitating mitigation of the depressurization before they are too hypoxic to function. These HAI programs are currently performed with either normobaric hypoxia (NH; lowered fraction of inspired oxygen (FiO_2_) to reduce the partial pressure of inhaled oxygen (PiO_2_)) or hypobaric hypoxia (HH; decrease in barometric pressure to reduce the PiO_2_). Whether neurophysiological differences exist between these two forms of hypoxia is not known.

Evaluating brain activity in the alpha frequency band (8–12 Hz) provides a convenient probe of neurophysiological responses to hypoxia. Previous studies have found that brain activity in the alpha frequency band is susceptible to hypoxia^2–6^, and alpha oscillations have also been reliably linked to attention control, such as in covert visuospatial attention tasks^7,8^.

Insights into neurophysiological processes supporting cognition can be gleaned from evaluation of EEG responses during attentional tasks. In particular, covert visuospatial attention, which is our ability to deploy attention to specific locations without moving our eyes, allows for enhanced processing of objects that are expected to appear in the covertly attended location, while supressing items located in the unattended/ignored visual locations^9^. This suppression of objects in the unattended location has been found to occur after attention has been deployed, but before target onset and is due, at least in part, to increased alpha frequency band activity^7,8,10^. Covert attention is a function of the visuospatial attention system^11^, and recruits general attention processes. Attention is a critical component of cognition^12^, and related to other vital cognitive processes such as working memory^13^. Thus, covert attention provides an excellent window into cognition and underlying neurophysiological processes essential for pilots to function. Covert attention tasks also allow investigation of alpha oscillations which are critical for inhibitory processes^8,14^ and cognitive control across numerous cognitive contexts^15,16^. Crucially, covert attention has been previously shown to be impacted by hypoxia^2–6^.

As well as serving as a gating mechanism for attention control, together with related processes such as visual working memory, activity in the alpha frequency band is also greatly impacted during hypoxia. Alpha power effects during hypoxic resting state conditions depends on whether the participant’s eyes are open or closed^6^, with increases in alpha power in hypoxia during eyes-open resting states^6^, and decreases during eyes-closed resting states^3,4,6^. This is interesting because during typical environmental conditions the opposite response occurs. An increase in alpha power was found during a flight simulation task while the participants were hypoxic^17^. Alpha power and oxygenation are linked, whereby alpha power is negatively correlated with the function magnetic resonance imaging blood oxygen level dependent signal response^18–21^, and oxygen saturation (SpO_2_) level^2^. A previous study on the impact of NH on visuospatial attention found an increase in alpha power over the right occipital and parietal areas, independent of cue location^22^. The increase in alpha power over the right hemisphere during hypoxia was interpreted by the authors as sustaining attention-orienting to cope with the hypoxic impairment. Collectively these data highlight that alpha power is modified by both attentional processes and hypoxia.

The beta frequency band (~ 13–30 Hz) has also been implicated in hypoxia and attention. Specifically, the beta frequency band serves to sustain visual attention, as beta power has been found to increase only before correct responses during a visual spatial discrimination attention task^23^, increased beta power is also associated with increased alertness represented by faster responses to visual stimuli during a visual spatial discrimination attention task^24^, and also as may serve as a compensatory mechanism of endogenous top down modulation to redirect attentional resources back toward the attentional task during psychosocial stress^25^. Lower-beta power (12.5–18 Hz) was found to be increased in the right superior frontal gyrus during eyes closed resting state with an acute hypoxic dose of 12.7 kPa PiO_2_ (95.26 mmHg)^26^. Similarly, beta power was found to increase during eyes open and eyes closed resting state with a 10% oxygen mixture^6^. Beta power was reported to decrease during button press reaction time studies at 2700 m for an hour^27^ and during a 12 h overnight stay at 4000 m^28^. For longer hypoxic exposures where acclimatization may have occurred, beta power increased during resting state recorded at 5050 m after a 14 day trek^29^, increased during resting state at 3800 m after 30 days^30^, and conversely decreased during resting state after 32 h at 3600 m and 6 days at 4300 m when compared to sea level^31^. Beta power decreases were also found to be correlated with decreased cognitive and flight performance during a NH flight simulation study at 7620 m (25,000 ft) and 6096 m (20,000 ft)^5^. In individuals with obstructive sleep apnea (OSA), which leads to intermittent hypoxia during sleep^32^, awake resting state recording show increased beta power^32,33^, with awake beta power correlated with nocturnal hypoxemia indices^34^. Significantly decreased beta power was found in severe compared to moderate OSA suffers during wakefulness, and men exhibited decreased beta power during sleep when compared to women^35^. Beta power has not been studied as extensively as alpha power in visuospatial attention and hypoxia research, yet these data suggest it plays an important role in both attention and hypoxia.

Debate still exists in the literature as to whether NH and HH are physiologically different, together with a knowledge gap regarding whether cognition, and the neurophysiological processes that support it, differs between these forms of hypoxia. Standardization of study methods is a major limitation in comparing NH and HH (e.g., humidity, sample size, length of hypoxic exposure) rendering discrepant study results difficult to reconcile^36^. For example, SpO_2_ is reported to be similar in NH and HH^37–41^, and yet also lower in HH than NH^42,43^. Minute ventilation is reported to be lower in HH^37,38,41,42,44^, and equivalent between HH and NH^43,45^. End tidal carbon dioxide levels (ETCO_2_) are noted to be similar in NH and HH^41–43^, higher in HH^37^, and lower in HH^38^. Breathing rate is reported to be greater in HH^42,43^, lower in HH^41,44^, and equivalent between NH and HH^37,38,45^. Lower tidal volume is reported in HH^37,41–44^. Heart rate variability is reported to be higher in HH^44^ or equivalent^46^. Blood pressure is reported to be higher in NH^41^, and also equivalent between NH and HH^37,45^. Furthermore, cerebrovascular reactivity to CO_2_ is more sensitive in HH than NH^47^, and hypobaria has a specific effect of decreased baroreflex sensitivity^48^. Postural stability is also more significantly impacted by HH, suggesting that hypobaria leads to the difficulties in balance reported at altitude^49^. Meta-regression analyses focusing on the effect of hypoxia on cognition found that hypoxia negatively impacted cognition, and that low arterial partial pressure of oxygen (PaO_2_) (35–60 mmHg) was the key variable in cognitive performance whether the study was performed in HH or NH^50^. Given the lack of standardization in study methods, and various cognitive tests utilized in cognitive studies, there is a need to further explore whether NH and HH are similar.

How neurophysiological dynamics supporting cognition are impacted by hypoxia remains poorly understood, together with whether NH and HH are distinct in their impact on these processes. To address this, we recorded EEG alongside physiological data while 13 participants performed a covert visuospatial attention control task during normobaric normoxia (NN), NH and HH conditions inside a hypobaric chamber. Estimates of neurophysiological activity were derived throughout brain source-space, and induced and evoked activity was calculated for canonical neurophysiological frequency ranges. We hypothesized that induced alpha and induced lower-beta power would be increased in the hypoxia conditions, with a greater increase in alpha power in HH. We hypothesize there will be greater increases in alpha power during HH because of the prior reports of lower SpO_2_ during HH^42,43^, and the negative correlation between SpO_2_ and alpha power^2^. Due to the novelty of our data set, together with the relatively scant knowledge in this field, we also conducted more exploratory analyses of induced and evoked power at other canonical frequency ranges. To our knowledge this is the first study to directly compare EEG activity between NH and HH during cognitive processing.

## Methods

### Participants

All participants provided written informed consent before participating in the study. The Simon Fraser University Research Ethics Board approved our study (#20160635), and the experiment was conducted in accordance with the Declaration of Helsinki. We recruited a sample of 22 participants from Simon Fraser University and the local community. Thirteen participants (5 female, age 26.1 (SD = 4.0) years, height 169 (SD = 9.7) cm, BMI 24.8 (SD = 3.1) kg/m^2^) successfully completed all three conditions and were used in the EEG analysis. Due to time constraints with using the hypobaric chamber, we were unable to record the female participants at similar phases of their hormonal cycle. Note that we had to exclude three participants because their SpO_2_ decreased below our SpO_2_ cut-off necessitating termination of the test, two participants due to a technical issue with our recording equipment, one participant due to a later disclosed psychiatric disorder, and one participant who had an adverse reaction to hypoxia (syncope). Additionally, two participants withdrew from the experiment after the first condition.

### Experimental conditions

This study employed a single-blind randomized design in which each participant completed three conditions, with each condition lasting up to 25 min. All three conditions were conducted inside a hypobaric chamber located 334 m above sea level, with at least 48 h between exposures. During the HH condition, participants were exposed to a simulated altitude of 3962 m (13,000 ft) and breathed the ambient air (21% oxygen, PiO_2_ = 87.2 mmHg) through a mask. We selected this hypoxic dose in accordance with Canadian Aviation Regulations, which limit hypoxic exposure in an unpressurized aircraft to less than 30 min, with cabin pressurization equivalent to 3962 m (13,000 ft). In the NH condition, the hypobaric chamber’s pressure remained unchanged, but participants breathed a hypoxic gas mixture calculated to elicit a PiO_2_ equivalent to the HH condition (12.8% oxygen, PiO_2_ = 87.2 mmHg). The NN condition also involved no change in pressure in the hypobaric chamber, and participants breathed the ambient air through a mask (21% oxygen, PiO_2_ = 143 mmHg). Participants wore a mask in all three conditions to ensure that they were blind to the experimental condition. For safety reasons, it was not possible to blind the inside tender.

#### Hypobaric chamber specifications

**Figure 1:**
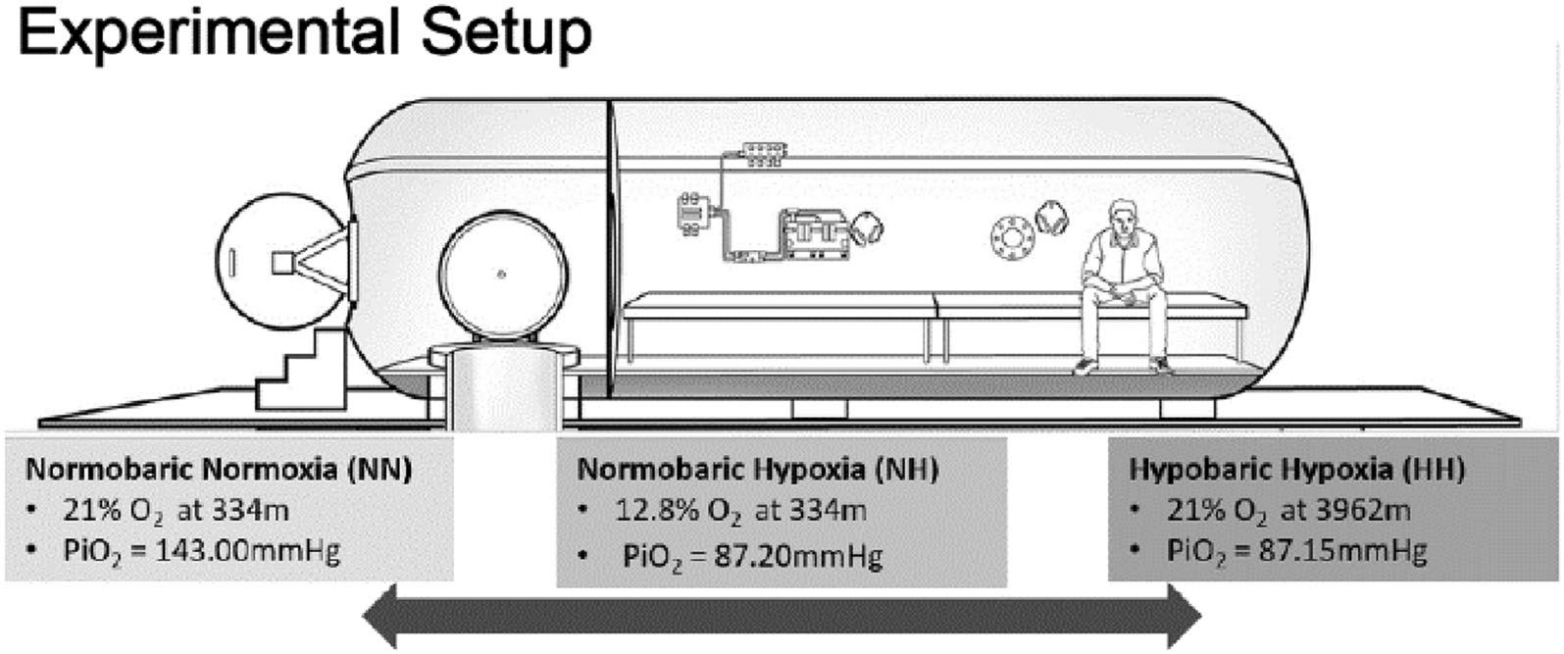
The experimental setup. All three conditions were recorded in the hypobaric chamber with similar PiO_2_. Adapted from Hutcheon et al., (2023).

The chamber used in the current study was constructed according to Pressure Vessels for Human Occupancy-One standards. The chamber is a triple-lock, multiplace Class “A” Hypo/Hyperbaric complex that contains an entry lock, main lock, and wet pot. Note that the wet pot, located beneath the chamber, was not used in the present study and accordingly the hatch depicted that leads to it was closed (Fig. 1). The chamber has a maximum working pressure capacity of 30 ATA (445 PSI) and a minimum working pressure of 0.01 ATA (0.15 PSI). The overall length, width, and height of the chamber are 7.3 m, 3.86 m, 2.2 m, respectively. All gas mixtures were delivered inside the chamber, and the participants wore modified masks (V2 mask Hans Rudolph Inc) throughout the experiment for all three conditions.

### Testing procedure

#### Participant instrumentation

Participants were tested at either 9 am, 12:30 pm, or 3:30 pm. First, participants were fitted with an EEG and brought into the hypobaric chamber. Once in the hypobaric chamber, participants had an SpO_2_ monitor (Nonin 7500 Pulse Oximeter) fitted to the left index finger for continuous recording. Beat-to-beat blood pressure was recorded with a finger photoplethysmographic device on their left middle finger with the height correction unit applied at heart level (Portapres model 2, TNO-TPD Biomedical Instrumentation, Amsterdam, The Netherlands). In one participant blood pressure data were excluded due to poor signal quality. A 2-MHz transcranial doppler ultrasound probe (DWL Doppler-Box, Compumedics) was placed over the right transtemporal window for continuous middle cerebral artery velocity recording, with the probe fixed in position by sliding it under the EEG cap. Cerebral blood flow velocity data were not analyzable in four participants due to movement of the ultrasound probe during testing. ETCO_2_ and ETO_2_ was recorded using an infra-red analyzer (O2Cap; Oxigraf Inc., Mountain View, Ca USA). End tidal gas data were excluded in two participants due to technical error (improper connection between mask and infra-red analyzer). All cardiovascular recordings were sampled at 1 kHz using an analog-to-digital converter (Powerlab 16/30; AD instruments, Colorado Springs, CO, USA). Participants sat on a bench with their eyes 60 cm away from a computer screen (Fig. 1.). Once sitting, the modified mask was put on the participants; however, it was not yet connected to a gas mixture (participants breathed ambient chamber air through the mask).

#### Experimental protocol

Prior to the commencement of the experiment, we connected the modified mask to the gas mixture, namely, a hypoxic gas mixture in the NH condition or ambient air in the NN or HH condition. A researcher located inside the chamber manually controlled the flow rate of the breathing gas to match the participant’s breathing rate, ensuring that the bag was never overfilled or collapsed on itself. Participants were instructed to sit still and not to talk during the entirety of the experimental protocol.

Participants first completed five minutes of an eyes-open resting state-recording before beginning the visuospatial attention task, with the results from that experiment stated in another publication^2^. Data were then collected during a visuospatial attention task lasting up to 25 min^7,8^, in which participants maintained fixation on a central cross. An arrow cue appeared, replacing the cross, prompting the participants to covertly attend to a box either left or right (14.38° visual angle laterally) from the fixation cross. The arrow cue (1.62° × 1.62° visual angle) was presented for 100 ms, followed by the fixation cross (1.29° by 1.29° visual angle). A target (“+” or “x”, with 50% probability) then appeared for 100 ms in either the left or right box, with a 50% probability, 1000–1200 ms after cue onset. Participants clicked a mouse button with their right hand if they detected a “+” in either location (cue or uncued box), and made no response if they detected a “x”. The targets appeared in a box (1.79° × 1.79°) 6.2° below the fixation cross. This experimental paradigm was adapted from previous studies^7,8^, which demonstrated changes in alpha power during visuospatial attention control. Following the completion of the experimental condition, participants were asked if they believed they had completed the HH condition and instructed to answer “yes”, “no”, or “not sure”. This was done to assess the effectiveness of the experimental blinding. Consideration of the efficacy of blinding was possible in 16 participants. Note that if participants’ SpO_2_ level dropped continuously below 80%, or if their systolic blood pressure dropped by 40 mmHg from the beginning of the recording, the participants were placed on 100% oxygen and the experiment was stopped.

### EEG recording

We recorded EEG with an EEG-Cap (BioSemi) system encompassing 64 Ag–AgCL active-electrodes (ActiveTwo) arranged on the participant’s head in accordance with the international 10–20 system. The electrodes were filled with Signa gel (Parker Laborities INC.) to ensure a stable connection. The electro-oculogram was recorded over the left eye with one electrode 1 cm form the outer canthi of the left eye and the other electrode above the left eye. The EEG signals were amplified, filtered, and digitized using the ActiveTwo AD-box (Biosemi) at a sample frequency of 512 Hz.

### EEG preprocessing

To preprocess EEG, we utilized the Fieldtrip toolbox^51^ in MATLAB 2019b, following the methods established in our previous research^2^. Statistical analyses were performed in MATLAB 2019b. First, we applied a 1 Hz high-pass filter, visually inspected each channel for excessive artifacts. The data were then referenced to the average across all electrodes. We then identified the triggers for left and right cue, and segmented the data into 1200 ms epochs with a 200 ms baseline, resulting in 560 trials in total per subject (280 for left cue and 280 trials for right cue). Any epochs containing significant muscle artifacts were visually identified and removed from further analysis. A 55 Hz low-pass filter was applied to the data to include lower gamma oscillations and remove any residual high frequency noise, including power line noise at 60 Hz. Next, eye blinks and saccades were first visually identified and then removed with Independent Component Analysis^52,53^. Any channels that were previously removed were interpolated based on surrounding channel data.

### Estimation of neurophysiological activity in brain source space

To reconstruct source dynamics for each participant we followed the methods established in our previous research^2^. A template head model was created using the standard Boundary Element Method volume conduction model^54^ with our appropriate electrode positions applied to the scalp of the head model. A Linearly Constrained Minimum Variance beamformer^55^ was used to define a set of weighting coefficients (spatial filter) on a 15 × 15 × 15 mm grid based on our head model and EEG data. The covariance matrix was regularized with a 5% diagonal loading. As such, we reconstructed neural activity at 590 locations throughout brain space.

### Event-related EEG responses

In our study, EEGs were recorded under an event-related experimental design. Time-locked neural activity was assessed with two measures of event-related activity: evoked and induced power of event-related EEG oscillations. In both cases, we started with time–frequency decomposition of EEG source dynamics. Specifically, we considered EEG time series, separately, for each trial and each source. We applied a complex Morlet wavelet transform to decompose the original source dynamics into the time- and frequency-specific EEG oscillations at 30 frequency points, equidistantly spaced on a logarithmic scale ranging from 4 and 55 Hz. As such, we calculated the phase and amplitude for each frequency and time point, representing the analytic signal of EEG oscillations for each trial, including the baseline.

We defined evoked power as time- and frequency-specific EEG activity that was both phase- and time-locked with respect to the event onset across trials. To estimate evoked power, we averaged frequency-specific EEG signals across trials, while taking into account both the phase and amplitude of EEG signals. Specifically, we first averaged the EEG analytic signal across all trials (left and right cue together), and then computed the absolute value of the resulting analytic signal to obtain the estimate of evoked power.

Evoked power analysis is a contemporary approach comparable to traditional event related potential (ERP) analysis. ERPs involve voltages time-locked to a stimulus, which are averaged over epochs to remove background EEG activity considered irrelevant to the task or mere “noise”^56^. However, this background activity reflects neuronal activity oscillating at various frequencies that contribute to event processing. Analyzing these time–frequency data can provide a more comprehensive understanding of the neural processes that are time locked to an event^56,57^. Induced power was estimated as time- and frequency-specific EEG activity that was only time-locked, but not necessarily phase-locked, with respect to the event onset across trials. To compute induced power, we first computed the absolute value of the analytic signal (time-specific wavelet power), separately for each trial, and then averaged the resulting signal across trials.

We analyzed the induced and evoked power of brain rhythms across five canonical frequency bands: theta (4–7 Hz), alpha (7–13 Hz), lower-beta (13–21 Hz), higher-beta (21–30 Hz), and lower gamma (30–55 Hz). From the wavelet decomposition, each frequency band was represented, on average, by six wavelet frequencies. To obtain the baseline-corrected power estimates, we first calculated the median value of the power estimates across time points belonging to the baseline period for each wavelet frequency. Then, we subtracted the baseline’s median power from the power estimates of the task period. Finally, we averaged the baseline-corrected power estimates across frequency points within each canonical frequency band. This resulted in 30 matrices of power estimates (two measures of event-related synchronization; three conditions; five frequency bands) per participant, with each matrix comprising 590 × 512 data points (590 brain locations and 512 time points, representing the first second of the task period).

### EEG statistical analysis for differences across conditions

To compare induced or evoked power across conditions, separately for each canonical band, we applied Mean-Centered Partial Least Squares (PLS) analysis. PLS is a non-parametric multivariate statistical technique widely used in neuroimaging^58,59^. Mean-Centered PLS serves as a multivariate technique that isolates latent variables responsible for the covariance between neural activity and predefined design variables, such as the relationship between signal power and pre-specified condition contrasts. This is conceptually similar to principal component analysis (PCA). Originating in the field of computational statistics, PLS was initially formulated as a methodology aimed at maximizing the explained covariance between two sets of variables through a minimal set of uncorrelated latent vectors^60^. It is alternatively known as “projection to latent structures”^61^. In the context of neuroimaging, we adhere to the terminology and approach of PLS as it leverages Singular Value Decomposition (SVD) of covariance matrices to robustly identify pertinent latent variables, which is further tested with permutation and bootstrap tests^59^. Krishnan et al.^58^ presented a tutorial and review on PLS methods in neuroimaging.

We performed a mean-centered PLS analysis on each canonical frequency band for induced power, and similarly for evoked power. To prepare the dataset for PLS analysis, we consolidate all observations from participants and incorporate all EEG features simultaneously. The data structure is organized as a matrix, where the dimensions represent participants nested within conditions and a vectorized form of EEG features. Specifically, power estimates for all unique combinations of time points and channels are vectorized.

The data input for PLS was thus organized as participants within each condition times EEG features. Each EEG feature was an estimate of power (induced or evoked) for a unique combination of a brain location and time point (in total, 590 × 512 = 302,080 features). In each PLS analysis, the covariance matrix across participants between EEG measures and variables representing experimental conditions was decomposed into a set of latent variables, using a SVD, which can be viewed as a generalization of PCA in case of non-square matrices.

More specifically, in our application of Mean-Centered PLS to investigate spectral power variations across conditions, we apply SVD to decompose the covariance between power metrics and design variables across the participant pool. SVD yields three matrices: the left singular matrix, which encapsulates overall condition contrasts; the right singular matrix, which highlights feature saliences; and the diagonal matrix, where singular values are sorted in descending order, quantifying the variance explained by each condition contrast and feature salience.

In the realm of statistical testing, a contrast represents a linear combination of variables—in our case, conditions—designed to evaluate a specific hypothesis. To assess the statistical significance of each condition contrast, we utilize permutation tests that shuffle subjects across conditions^58^. A contrast is deemed significant if its original singular value in the diagonal matrix diverges substantially from those generated through permutation. We note that the contrasts we obtain are not pre-defined but are instead driven with a projection which explains the largest variance, similar to PCA. For each PLS, we report the first contrast, which explains the largest amount of variance of changes across conditions. Expressed in arbitrary units, each contrast, which is three-dimensional in our case, is accompanied by a single p-value that quantifies the statistical significance of the resulting model.

Additionally, we evaluate the robustness of each EEG feature’s contribution to the corresponding condition contrast through bootstrap testing^58^. Both permutation and bootstrap tests were based on 1000 random samples. From the bootstrap procedure, we obtain a vector of bootstrap ratio values for all unique combinations of time points and channels. We use the terms “bootstrap ratio values” and “z-scores” interchangeably. For visualization, these z-scores are transformed back into brain space, represented as a matrix of time points by EEG channels (590 × 512). The condition contrasts and z-scores must be interpreted in conjunction.

To interpret the directionality of effects represented by the overall contrast across conditions (that is, to know if EEG power is higher or lower in one condition compared to other conditions at a given brain location and time point), we had to consider PLS’s contrast and z-scores together. Large in magnitude positive z-scores directly support the contrast across conditions. Large in magnitude negative z-scores also support the contrast, but in a reserved way. For the purpose of visualization, each source-time map of z-scores (for each frequency band) was projected onto the cortical surface. To visualize the effects across time, for each brain location, we averaged the z-scores (as the median) across time points within non-overlapping, 100 ms long, time intervals throughout the one second task interval. Accordingly, we have 10 snapshots of z-scores across the entire brain.

### Statistical analysis of physiological measures

A 3-way repeated measures ANOVA comparing our 3 conditions (NN, NH, HH) was performed on the average (cleaned from artifacts) SpO_2_, ETCO_2_, ETO_2,_ systolic blood pressure, diastolic blood pressure and breathing rate. A Tukey post-hoc test was employed. The mean ± standard deviation were also calculated and reported for each condition. A statistical threshold of *p* = 0.05 was employed. For technical reasons, nine participants were included in the transcranial Doppler data analysis, 12 participants for the blood pressure analysis, 11 participants for the end tidal gas analysis, 13 participants for the SpO_2_ analysis, and 16 participant responses to the blind question were included. All statistical analysis for physiological measures were performed with JMP 16.

### Analysis of experimental blind results

The analysis of our experimental blind responses was conducted using SAS on Demand statistical software. To compare the mean responses of the three categories (Yes, No, Unsure) between different conditions (NN, HH, NH), we applied a generalized linear model with a multinomial distribution and cumulative logit link function, using Proc Genmod. The effect of condition was considered a fixed effect factor in the model. Since multiple measurements were taken on each subject, the effect due to subjects was also accounted for. To compare multinomial proportion responses between pairs of condition levels, we applied Post hoc tests using a Tukey Kramer adjustment. Additionally, bivariate tables were generated to characterize the proportional breakdown between condition levels. Figure 8 presents the frequency of responses for visualization purposes only and was not included in the statistical analysis.

### Ethical approval

The study was approved by the Simon Fraser University Research Ethics Board and all participants provided written informed consent.

## Results

### SpO_2_ and ETO_2_ was significantly higher in NN

**Figure 2:**
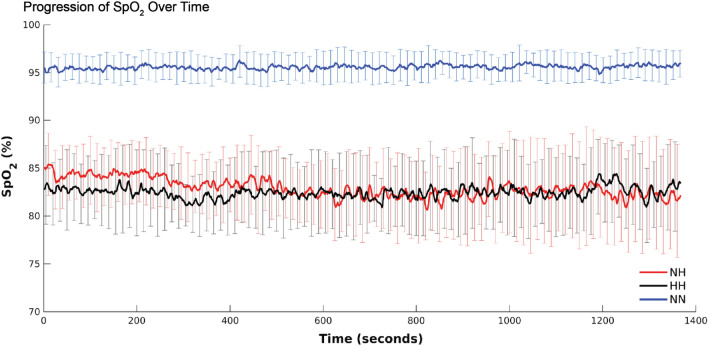
SpO_2_ levels over time. SpO_2_ for NN was significantly higher than NH and HH, with NH and HH not differing from one another.

The average SpO_2_ values were 95.7 ± 1.4% for NN, 82.4 ± 3.2% for HH, and 82.9 ± 3.3% for NH (Fig. 2) which were to be expected at our chosen altitude of 3962 m^62,63^. Results from our repeated measures one way ANOVA show that SpO_2_ during NN was significantly greater than in NH and HH, but NH and HH did not differ from one another (*F*_2,24_ = 189.5,* p* < 0.001). The average ETCO_2_ values were 25.64 ± 5.4 mmHg for NN, 27.0 ± 4.3 mmHg for HH, and 27.8 ± 4.2 mmHg for NH with no significant difference between conditions. The average ETO_2_ values were 113.1 ± 6.7 mmHg for NN, 59.6 ± 5.7 mmHg for HH, and 63.8 ± 5.5 mmHg for NH. The NN condition was significantly higher than both NH and HH, which did not differ significantly from each other (*F*_2,20_ = 373.5,* p* < 0.001). The average breathing rate per minute was 15.5 ± 3.4 for NN, 15.1 ± 2.9 for HH, and 14.9 ± 3.5 for NH with no significant difference between conditions. The average systolic arterial blood pressure values were 116.5 ± 9.8 mmHg for NN, 114.9 ± 18.3 mmHg for HH, and 118.9 ± 18.3 mmHg for NH with no significant difference between conditions. The average diastolic arterial blood pressure values were 60.6 ± 8.7 mmHg for NN, 57.4 ± 14.3 mmHg for HH, and 62.6 ± 12.6 mmHg for NH with no significant difference between conditions. The average MCA CBF velocity was not significantly different between conditions (NN: 42.5 ± 2.7 cm s^−1^; HH 36.5 cm s^−1^; NH: 38.4 ± 9.0 cm s^−1^).

### Induced alpha power was significantly higher in NN and induced Lower-beta power was significantly higher in HH

**Figure 3:**
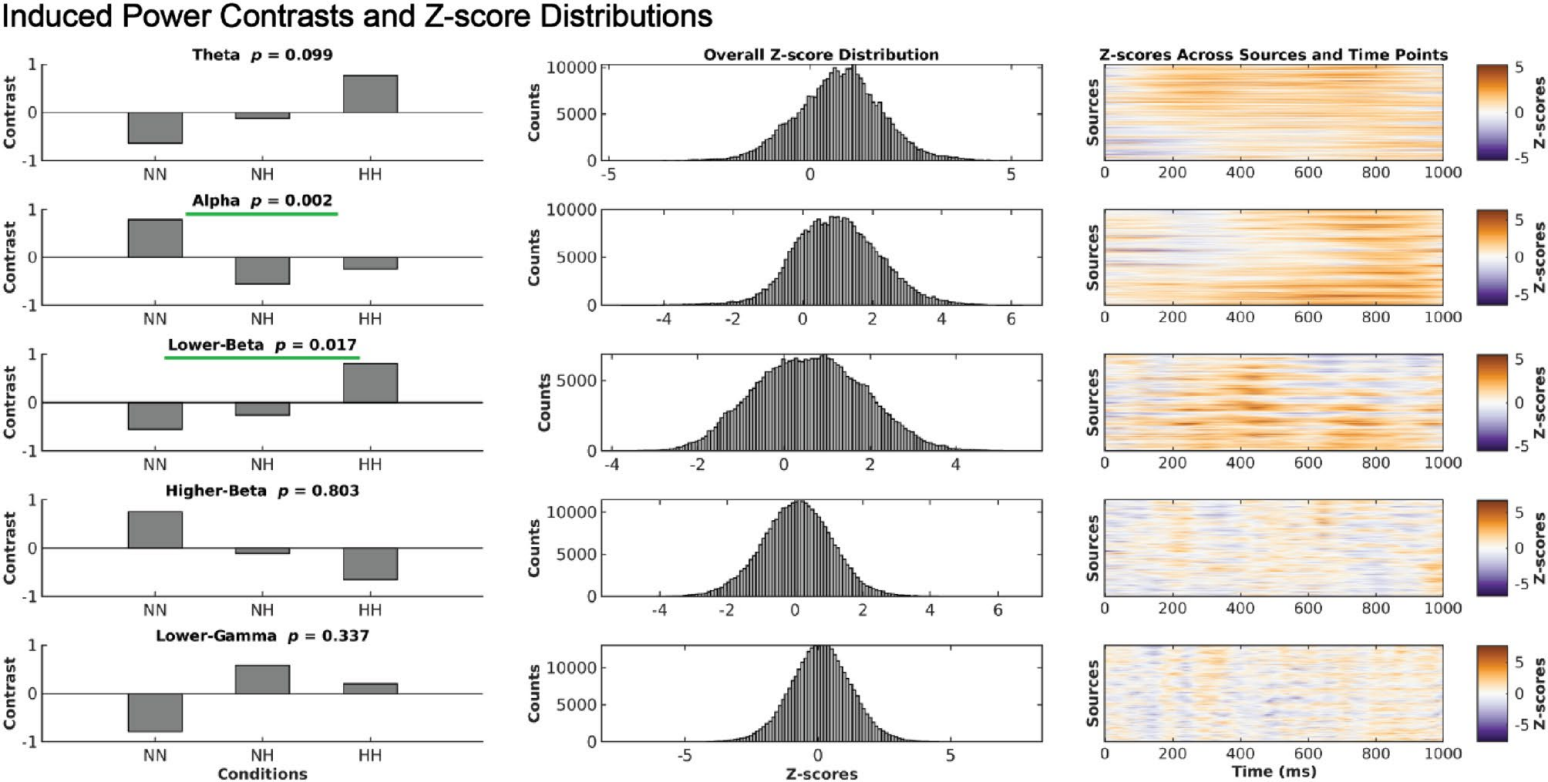
Increased induced alpha power during NN and increased induced lower-beta during HH. Our statistical analysis revealed significant differences in induced power of alpha and lower-beta oscillations among the NN, NH, and HH conditions. Specifically, NN exhibited higher induced alpha power (*p* = 0.002) compared to NH and HH, while HH exhibited higher induced lower-beta power (*p* = 0.017) compared to NH and NN. The contrasts in the panels on the left exhibit the pattern that explains the most variance in overall differences across conditions, as determined by PLS. The histograms in the middle column display the overall distribution of z-scores, which are shifted toward positive values for both alpha and lower-beta. These z-scores represent the robustness of the contribution of each EEG feature (i.e., each source, time and frequency point) to the overall contrast. The heatmaps in the panels on the right exhibit the same z-scores as in the histograms, but across source and time points. Positive z-scores directly support the overall contrast. Specifically, positive z-scores, shown in red, indicate increased induced alpha power in the NN condition compared to the NH and HH conditions, and increased induced lower-beta power in HH compared to NN and NH.

**Figure 4:**
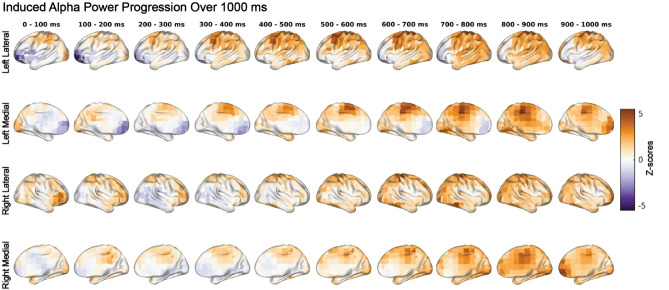
Dynamics of induced alpha power during the cue-target interval with median z-scores in 100 ms time bins. Positive z-scores indicate higher induced power of alpha oscillations during NN, whereas negative z-scores indicate higher alpha power during hypoxia (NH or HH). During the first 300 ms, HH and NH exhibited higher induced power of alpha rhythms in the left prefrontal cortex. This effect disappeared after 300 ms. In contrast, NN exhibited higher induced alpha power over the parietal regions, corresponding to decreases in induced alpha power during NH and HH.

**Figure 5:**
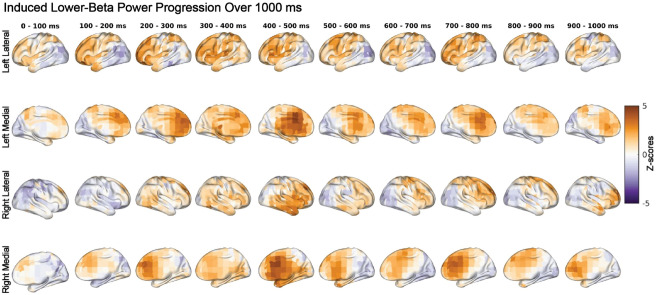
Dynamics of induced lower-beta power during the cue-target interval with median z-scores in 100 ms time bins. Positive z-scores indicate increased induced lower-beta power during HH, while negative z-scores indicate increased induced lower-beta during NN. We observed a widespread increase in induced lower-beta power during the HH condition. The left hemisphere exhibited an increase in induced lower-beta power first over the frontal lobes that then progressed over the parietal regions.

We observed significantly higher induced alpha power during NN compared to HH and NH conditions (*p* = 0.002), as shown in Fig. 3. The maps of z-scores projected to the brain surface in Fig. 4 revealed widespread higher alpha power in the NN condition that was strongest over parietal and central locations from 300–1000 ms in the left hemisphere, and 500–1000 ms in the right hemisphere. The NH and HH conditions exhibited higher alpha power centered in the left prefrontal cortex during 0–300 ms of the cue target interval. Furthermore, we found a significant increase in induced power of the lower-beta oscillations during HH compared to NN and NH (*p* = 0.017). This pattern also indicated increased induced lower-beta power in NH than NN. The cortical surface projections of z-scores in Fig. 5 showed increased induced lower-beta power primarily in the left hemisphere over frontal-parietal regions during HH, which first appeared anteriorly and progressed posteriorly to the parietal region. The temporal and occipital regions showed a slight increase in induced lower-beta power for the NN condition.

### Evoked higher-beta power was significantly higher in HH

**Figure 6:**
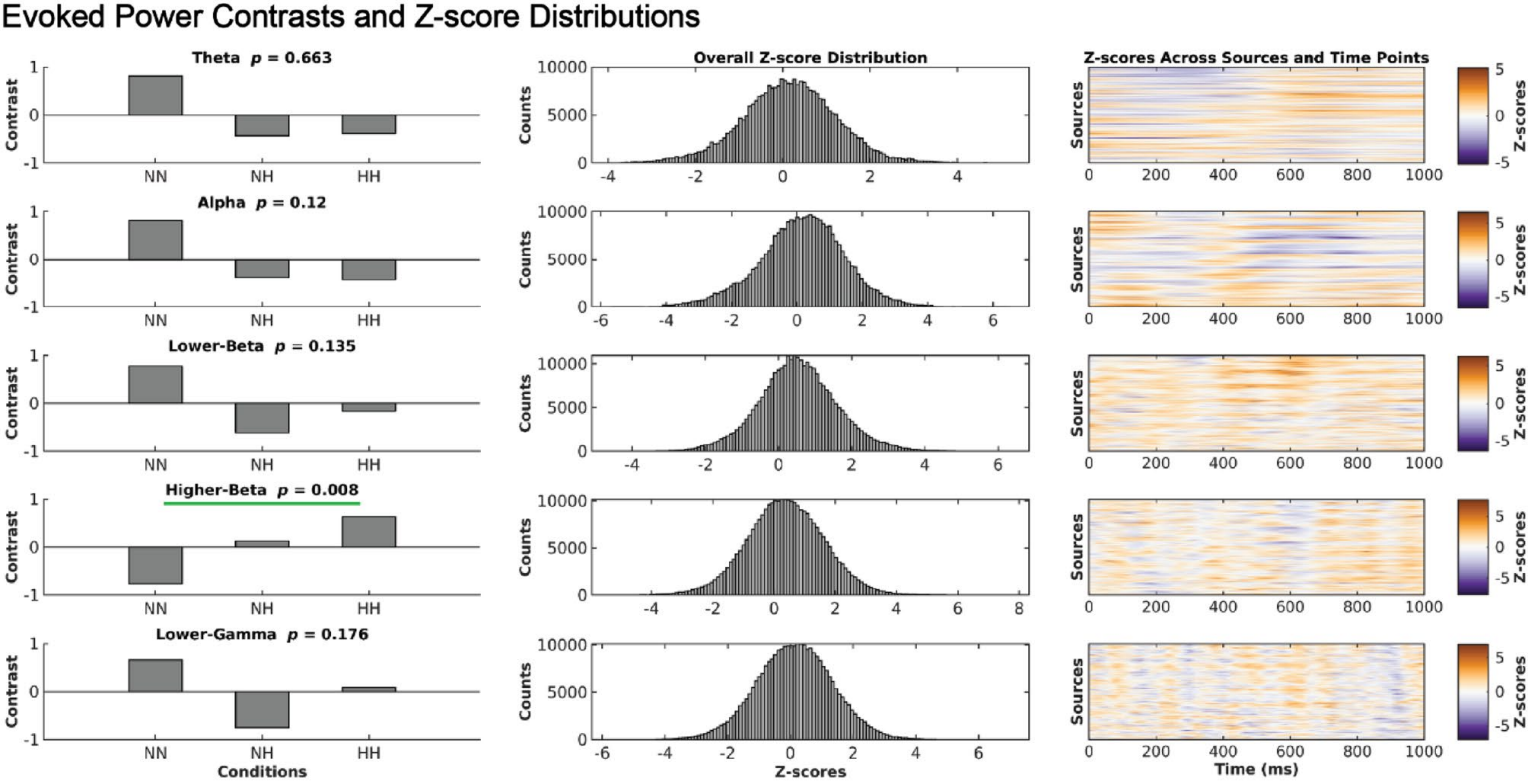
Evoked power of higher-beta rhythms is significantly higher in HH relative to NH and NN. The panel on the left shows the patterns that explain the most variance in conditional differences, as determined by PLS. The middle panel shows the overall distribution of z-scores (across all EEG features, across all time points and sources). The corresponding distribution has a rightward shift for higher-beta. The z-scores reflect the robustness of the contribution of each EEG feature (each source and time point) to the overall contrast. The right panel exhibits the same distributions of z-scores, as in the middle panel, but across all sources and time points. The overall distribution of z-scores associated with evoked higher-beta power was skewed toward positive values, indicating the most robust effect. Specifically, positive (red) z-scores indicate significantly increased evoked power of higher-beta rhythms recorded in the NH condition compared to the NN and HH conditions (*p* = 0.008).

**Figure 7:**
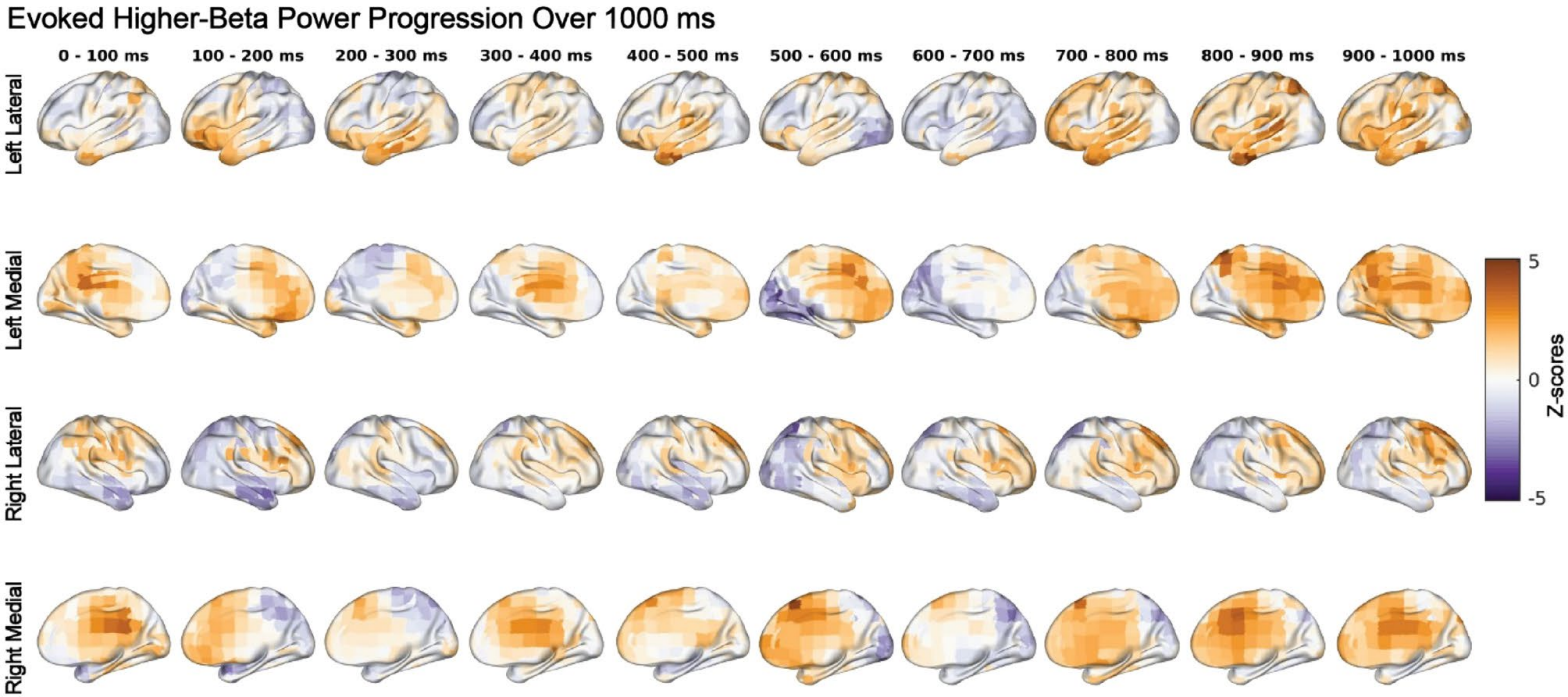
Dynamics of evoked higher-beta power during the cue-target interval with median z-scores in 100 ms time bins. Positive z-scores indicate an increase in evoked higher-beta power during HH, and negative z-scores an increase in evoked higher-beta power during NN. We observed a widespread increase in evoked higher-beta power during the HH condition. Evoked higher-beta evoked power was greater over frontal regions.

Our results demonstrated a significant increase in evoked higher-beta power (*p* = 0.008) in the HH condition compared to the NN and NH conditions, as shown in Fig. 6. The cortical surface projections of z-scores in Fig. 7 revealed a widespread increase in evoked higher-beta power with increased evoked higher-beta power over the frontal regions that was less pronounced in posterior regions, with an almost anterior and posterior split. Evoked higher-beta power then increased over widespread cortical regions on the left hemisphere, while the right hemisphere exhibited a more frontal cortex localization.

### Efficacy of experimental blind question

**Figure 8:**
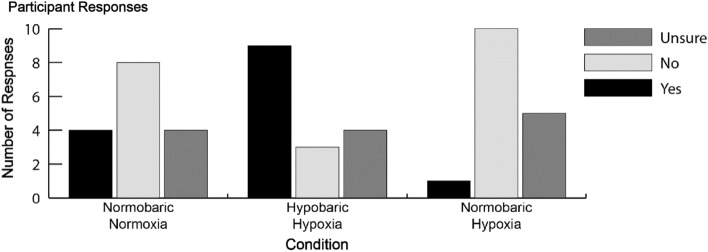
Participant responses to the question of whether they thought they completed the HH condition that day. The proportion of responses differed significantly between NH and HH, and NN and HH (*p* = 0.0006). Overall, participants appeared to guess correctly whether they were in the HH condition. However, if we combine the “No” and “Unsure” responses, only 56% of participants answered “yes” (9/16 responses) to the HH condition suggesting that our experimental blind was successful. Notably, the proportion of responses was similar between NN and NH, indicating that participants could discern that they were not in the HH condition but not that they were hypoxic. Figure 8 illustrates the sum of each response (yes, no, unsure) for each condition.

The tested generalized linear model yielded a statistically significant effect of condition (*p* = 0.0006), and no significant effect of participant. The post hoc analysis using Tukey–Kramer tests revealed significant differences in the proportion of responses between the control and HH conditions (*p* = 0.035) and between the HH and NH conditions (*p* = 0.0019), but no significant difference between the NH and control conditions. Upon combining the “no” and “unsure” responses, the visual analysis revealed that participants performed slightly better than chance in correctly identifying the HH condition. The distribution of responses across conditions is shown in Fig. 8.

## Discussion

We demonstrated that induced alpha power, induced lower-beta power and evoked higher-beta power were impacted by hypoxia. Induced alpha power was significantly decreased during hypoxia (no differences between NH and HH) while induced lower-beta power was impacted by hypobaria, with significantly greater power in HH compared to NH and NN (Fig. 3). These data indicate that atypical alpha may be sensitive to hypoxia but not hypobaric conditions while lower-beta is sensitive to hypobaria and hypoxia. Interestingly, the decrease in induced alpha power we observed during hypoxia was contrary to our hypothesis. This may reflect the novelty of our study. The lower induced alpha power during hypoxic conditions may be a dysregulation of visuospatial attentional control induced by hypoxia, as upward modulation of alpha power is related to the suppression of to be ignored information^7,8^. It appears that in the control condition participants were successfully able to increase alpha over to be ignored areas, while hypoxia led to a deficit in this system, whereby the brain during systemic hypoxia was unable to recruit alpha power to supress information in the to be ignored locations. Our exploratory analysis found beta power, both induced (Fig. 3) and evoked (Fig. 6), significantly differed between NH and HH. In both cases beta power differed in a stepwise fashion between conditions, indicating that NH caused an alteration of the beta-band response, and that the magnitude of this alteration was amplified in HH. This provides evidence that the impact of HH on neurophysiological processes supporting cognition may be greater than NH, even when PiO_2_ and SpO_2_ are the same. Of note, these differing EEG responses could not be explained by associated alterations in cardiovascular or respiratory control, as with the exception of the obvious differences in end tidal and arterial oxygen levels, there were no differences in respiratory, cardiovascular or cerebrovascular control between conditions. We were the first to compare EEG responses to NH and HH^2^, and we now extend and enhance these observations by directly comparing EEG responses to NH and HH during a cognitive task. To our knowledge only two previous studies^2,26^ have investigated hypoxia in brain source space with an EEG.

Alpha oscillations play an important role in cognition, and reflect inhibitory control underlying diverse processes including attention control^13^ and working memory^11^. The alpha frequency band plays a prominent role in attention orienting, as an increase in alpha represents a top-down preparatory mechanism for attention allocation to relevant areas^7,8,14,22^. Our results show that induced alpha power was decreased during both NH and HH when compared to NN (Fig. 3), which suggests a hypoxia induced dysregulation of neurophysiological processes underpinning attentional orienting. Interestingly, the prefrontal cortex has been implicated in shifts of attention during spatial attention tasks^64^ and the increase in induced alpha power during NH and HH in the left prefrontal cortex (Fig. 4) may represent a hypoxic compensatory processing mechanism to recruit extra attentional resources due to cue expectation. Lowlanders living at high altitude have been found to have reduced P300 amplitude during a visuospatial task when compared to lowlanders at low altitude, which suggests that the participants living at high altitude have reduced attentional resources^65^. Even though the previous study examined individuals acclimatized to living at high altitude, and our study focused on individuals given an acute dose of hypoxia, our results were similar. A decrease in attentional resources due to hypoxia may be evident with a decrease in alpha inhibition of to-be-ignored visual information. Since this inhibitory role of alpha is not limited to attention orienting per se, but rather reflects a more general process relevant for selection of information for further processing, these effects of hypoxia on attention may impact other cognitive domains including working memory and alertness^13,66–68^.

Induced (Fig. 3) and evoked (Fig. 6) spectral power changes due to hypoxia observed in the present study highlight the controversy in the field, and difficulty in comparing studies. Our decrease in induced alpha power was similar to that found in previous studies that employed eyes-closed resting state^3,4^ and a flight simulation program^5^. A study employing a similar attention paradigm to ours found an increase in alpha power during NH when compared to NN^22^; however, they looked at power at specific electrodes while we utilized a source space analysis. It may be that during eyes open resting state we see an increase in alpha; however, once an eyes-open task begins hypoxia induced deficits may occur as the brain is unable to properly allocate alpha for the specific task demands resulting in a decrease in alpha power. Beta power has been found to increase during eyes-open (all frequency bands increase) and eyes-closed (alpha power decrease, all other frequencies increase) resting state^6,26^. To our knowledge we are the first to highlight induced lower-beta power and evoked higher-beta power as key frequency bands in differentiating between both forms of hypoxia. Previous studies have highlighted alpha power as a hypoxia sensitive frequency band^3,4,6^.

Our a priori hypothesis for EEG data was focused on induced alpha activity. Given that source-resolved EEG studies comparing NH and HH are rare, we also performed data-driven analyses of evoked and induced power across multiple canonical frequency ranges. This revealed that induced lower-beta was impacted in a stepwise manner (Fig. 3) in which NH deviated from the neural response recorded in NN, and HH showed a similar alteration which was significantly greater in magnitude. This indicates that NH and HH are distinguishable in their impact of neurophysiological dynamics during cognitive processing, with HH eliciting a larger alteration of this brain response even when SpO_2_ levels (Fig. 2) were the same across the two hypoxia conditions. Evoked higher-beta power showed a very similar effect as well, which was much more statistically reliable (Fig. 6). Beta band power has been found in previous studies to play a role in visual attention, for example having been found to increase during the anticipation phase of a visual attention task in cats measured using intracortical electrodes^69^. A negative correlation between beta power and reaction time has also been previously reported, with increased beta power associated with decreased reaction time (increased alertness) over parietal regions during a spatial discrimination task^24^. A source space study comparing NN with NH with a PiO_2_ of 95.26 mmHg found an increase in beta-1 (12.5–18 Hz) in the right superior frontal gyrus, which the authors suggest may help with attention and arousal^26^. Hypoxia may have led to an alpha induced attentional deficit that was compensated through enhanced beta power. Another possibility is that the altered beta response directly reflects the effects of hypoxia and hypobaria, rather than compensation to it. Further research will be required to disentangle these possibilities.

Speculatively, the larger increase in beta power in the HH condition than in NH (Figs. 3 and 6) may reflect an effect of hypobaria due to the decreased air pressure in HH that was not present in NH. The decreased air pressure during HH may differentially impact airflow sensitive receptors in the nose, leading to potential disruption of respiration-modulated brain oscillations. In humans, nasal airflow is sensed by trigeminal cool thermoreceptors which respond to airflow by mucosal cooling^70^. In HH the air is less dense, which leads to a decreased work of breathing^71^, and an argument could be made that less dense air also may impact the thermoregulatory receptors as the lower density gas leads to less turbulent airflow with less of the airstream in contact with the mucosal wall and consequently reduced receptor activation. According to studies using mice, in mammals the olfactory bulb receives feedback information from specific G-protein coupled receptors that are sensitive to and triggered by airflow in the nasal cavity^72–74^ that are part of a respiratory network resulting in entrainment of brain oscillations with respiration. Similar to mice, a large respiratory network modulating oscillatory activity has been found in humans^75^, and nasal airflow information has been found to be processed alongside olfactory information at multiple stages in human olfactory processing^76^. Humans likely express a similar mechanism where airflow receptors connected to the olfactory bulb entrain neural oscillations following the respiratory rhythm, and then this sends this information to upstream areas as part of a large respiratory network encompassing cortical and subcortical regions (e.g., cerebellum) as a feedback signal^75^. These respiration-modulated brain oscillations were found in all analyzed frequency bands. In particular, beta power was greatest during onset of inspiration and was highly modulated in resting state networks including the dorsal attention network which is critical for spatial attention^75^. Beta power is the most strongly expressed frequency in task positive networks like the dorsal attention network, and is critical for communication with the dorsal attention network^77^. Cognitive tasks can be influenced by respiration, with better cognitive task (visuospatial attention and memory) performance during inhalation compared to exhalation^78,79^ alongside increased beta power during inhalation in the parietal region^78^. An increase in oscillatory theta, delta and beta band power was found with inspiration in the piriform cortex, with breathing entraining neural oscillations between olfactory cortex and limbic areas^79^. The parietal cortex is involved in attentional processes^80,81^, and increased beta power over the parietal cortex during inhalation at task onset suggests that inhalation was preparatory for incoming sensory information and to maintain attention^78^. Participants would opt for trials that coincided with their inhalation, and no difference was found between oral and nasal breathing in terms of visuospatial task performance^78^. Speculatively, although our breathing rates and ETCO_2_ levels were similar between NH and HH, the decreased air density in HH may have sent a disrupted airflow signal, altering the respiration networks. This may have made it easier for the participants to time inhalation to cue onset, as beta power increases were found with inhalations timed to cue onset during a visuospatial attention task^78^. We show an evoked higher-beta power increase during HH in the left inferior frontal cortex and temporal lobe (Fig. 7), which may be due to olfactory bulb and olfactory cortex activity of the respiratory network^78^.

The increase in evoked and induced beta power in the HH condition can, speculatively, be attributed to top-down control to shift attention towards our visual spatial attention orienting paradigm. Attention can shift between endogenous top-down control (internal, based on goals and expectations) and exogenous bottom-up processing (external, based on stimuli sensory properties)^81^. Increased beta power has been correlated with top down attentional control^82–84^ and has been argued to send top down influences to modulate low level sensory neurons^85,86^. Top down beta rhythms modulating early sensory processes have been supported by computational modelling^87^. Beta power was found to increase during psychosocial stress during an attentional task, suggesting that this increased beta power serves as a top down compensatory mechanism to shift attentional resources back toward the ongoing task and not toward internal stress related thoughts^25^. At the end of every condition, we asked the participant if they thought they had taken part in the HH condition, and this may have primed the participants for the later two conditions to try and guess which condition they were in, and some were accurately able to distinguish the HH condition (Fig. 8). Thinking that they are in the HH condition may induce stress in the participants and shift their attention endogenously as they internally debate whether they think this is the HH condition. Alternatively, they may have required more top-down resources to maintain task focus due to increased demand on neurological function in the HH condition unrelated to psychological stress. Given that attention modulates early sensory processing^88,89^, our higher induced lower-beta power may be an endogenous shift of attention back to our visuospatial task leading to a top-down modulation of bottom-up processing exhibited by our evoked higher-beta power increase (i.e., top down processes modulating early evoked sensory processes). Our increased beta power may then be a top-down compensatory mechanism to shift our participants attention back to the task.

For various reasons, not all participants completed the study, with an obvious impact on statistical power. Nevertheless, we were able to detect significant differences in neurophysiological responses based on alpha power during hypoxia, and in the case of beta power, differences between NH and HH.

There is always a risk that breathing through a facemask would influence rate and depth of breathing. Given that a mask was worn in all three conditions, however, it is not likely to have influenced our results. Of note, respiratory rates and end tidal carbon dioxide levels were similar in all three conditions, and suggest that participants were hyperventilating in all three conditions. This is expected in the NH and HH conditions, but not in the NN condition, and may reflect hyperventilation in the presence of the facemask and in anticipation of potentially experiencing a hypoxic stimulus. As expected in the face of hyperventilation, MCA CBF velocity was reduced compared to expected values in healthy controls^90^ but was similar in all three conditions, suggesting any alterations in the neurophysiological responses in the different conditions do not reflect differences in CBF. Our participants were heavily instrumented, and our instrumentation paradigm did not permit the additional measurement of eye movement with an eye tracker, so we cannot say for certain if the participants kept their fixation on the fixation cross during the covert attention task.

We directly compared neurophysiological responses during a cognitive task between NH and HH at a PiO_2_ of 88.2 mmHg with an EEG. We found lower induced alpha power during NH and HH when compared to NN, but no difference in induced alpha power between NH and HH (Fig. 3). We also showed greater evoked higher-beta (Fig. 6) and induced lower-beta power (Fig. 3) in HH than NH and NN. As expected, SpO_2_ (Fig. 2) alongside ETO_2_ was significantly higher in NN than HH and NH, with HH and NH not differing from each other, and other than our EEG findings, none of the other physiological recordings were able to differentiate HH from NH. Of note, some of our participants were also able to differentiate NH from HH at an equivalent PiO_2_. With this in mind we suggest that future HAI courses are performed in HH, as the neurophysiological stimulus is different to that of NH, and more closely approximates a depressurization event that may occur during flight. Of note, our study was performed with relatively mild hypoxia (3962 m), with HAI courses being performed with a severe hypoxic dose of 7620 m (25,000 ft). Further research is needed to elucidate the neurophysiological basis of this evoked higher-beta and induced lower-beta difference between the two hypoxic paradigms, and whether this difference is relevant to HAI courses.

## Data Availability

The data that support the findings of this study are available from the corresponding author upon reasonable request.
